# Poisoning with Oil of Bitter Almonds

**Published:** 1852-03

**Authors:** 


*Poisoning with Oil of Bitter Almonds.*—On Thursday, July 24th, Mr. William Carter, the Surrey Coroner, held an inquest of some hours duration, at the Queen's Arms Tavern, Spa Road, Bermondsey, on the body of Mrs. Sarah Spencer, aged twenty-six years, the wife of Mr. Spencer, the perruquier and perfumer, of King William Street, City, who died from the effects of prussic acid, at her private residence, No. 4, Spa Road. A great number of witnesses were examined, but the following are the short facts of the case, as detailed by the coroner: Some few weeks since, the deceased was confined with her first child, and ever since she has been in an exceedingly low and desponding state, but from what arising no one was able to form the slightest conception. She frequently spoke to her attendants of her unhappy state of mind, and more than once said that she should soon die. She also said that she was not like some other parties, or she would have died some time back, (alluding, as the witnesses thought, to her having taken poison on previous occasions.) On Monday last, she went out and purchased at the shop of Mr. Elkington, the chemist, of 10, Bamford Lane, Bermondsey, a drachm of the essential oil of bitter almonds, and a penny-worth of linseed meal. She made application for a quarter of an ounce, under pretence of wanting it to scent some pomatum, but Mr. Elkington refused to sell her a larger quantity than one drachm. On leaving the shop, she remarked that it was useless for Mr. Elkington to be so determined, for if she chose, she could get a small quantity at each shop in the neighborhood; and smiling, replied, with all his precautions, he could no t bottle up the Thames. She then repaired to her home, and the next morning her husband found her in bed in an insensible state. Dr. Paul, who had attended her in her accouchment, was sent for, and on his arrival found her suffering from the effects of prussic acid. Every thing was done to save her life, but without effect, and she died in less than half an hour. The jury having consulted, rendered a verdict of temporary insanity.—*London Pharm. Journal.*

